# Telemedicine – chances and challenges for medical genetics in Germany

**DOI:** 10.1515/medgen-2021-2057

**Published:** 2021-05-14

**Authors:** Johanna Tecklenburg

**Affiliations:** Institut für Humangenetik, Medizinische Hochschule Hannover, Carl-Neuberg-Straße 1, 30625Hannover, Germany

**Keywords:** telemedicine, virtual consultation, teleconsultation, future of care, telemedicine outcome

## Abstract

Telemedicine has been in practical use for many years, mostly within the context of model projects. The current Covid-19 pandemic has accelerated the process of implementing telemedicine in standard care. Numerous regulations, as well as complex reimbursement structures play a role in the application of telemedicine in medical genetics in Germany. Discipline- and technology-specific challenges complicate the integration of technical solutions into the medical genetic practice. In previous studies teleconsultations and virtual consultations in medical genetics have proven their value as indicated by high levels of satisfaction in the users and showing no inferiority to in-person consultation in terms of psychosocial outcome. The next years will bring an increasing demand for genetic counseling that can hardly be met by the limited number of specialists in Germany. In this context telemedicine can help to close these gaps in standard care while strengthening the field by ensuring comprehensive medical genetic care. The German medical genetics community is asked to actively shape the process of implementation by defining areas of genetic counseling that are suitable for telemedicine, by regulating access for physicians and by contributing to the renumeration structures.

## Introduction

As early as 2005 the World Health Organization has urged its member states to “draw up a long-term strategic plan for developing and implementing eHealth services in the various areas of the health sector” stating that “eHealth is the cost-effective and secure use of information and communications technologies in support of health and health-related fields” [1]. Telemedicine as part of eHealth is the use of information and communication technologies for various medical care concepts in which medical services are provided in the areas of diagnostics, therapy, and rehabilitation as well as in medical decision-making across spatial distances or time shifts [2]. The German Medical Association declared telemedicine to be a future task for the medical profession in 2010 and defined basic requirements and objectives for telemedical methods in 2015 (Table 1) [3], [4].

**Table 1:** Requirements and objectives for telemedical methods according to the German Medical Association.

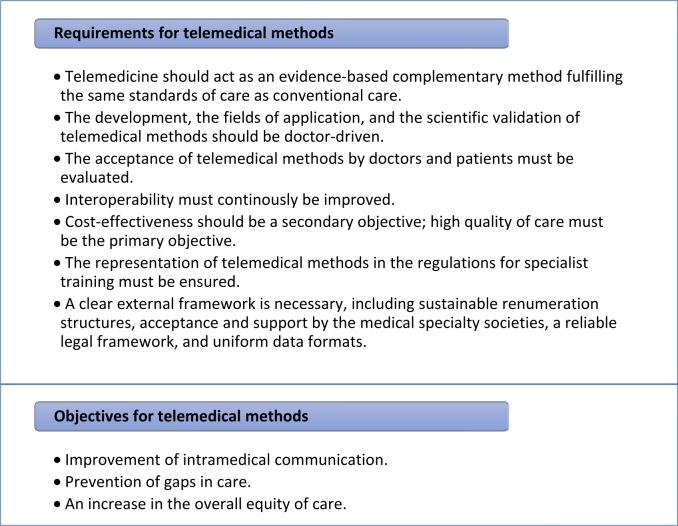

In the past, innovative concepts for the use of telemedicine in standard care in Germany have been implemented almost exclusively within the context of local model projects or new forms of care under the Innovation Fund of the Federal Joint Committee (Gemeinsamer Bundesausschuss). The German Federal Ministry of Health, however, has identified digitization as a central prerequisite for the successful advancement of our healthcare system and is pushing ahead with the implementation of telemedicine in standard care as part of its eHealth initiative. The Covid-19 pandemic and the resulting need for telemedical care has highlighted the immense potential of telemedicine for the further development of our healthcare system. A study by the German management consultancy firm Roland Berger has estimated the volume of the digital healthcare market in Europe to rise to €232 billion by 2025, an increase of almost 50 % compared to forecasts before the pandemic. The prognosis made for Germany expects a volume as large as €57 billion. The experts have assumed that the pandemic will accelerate the digitization process of the industry by approximately 2 years. The biggest winners will be platforms that link a large variety of applications and players [5]. We are, therefore, witnessing an accelerated and irreversible change in the field of telemedicine, where no longer doctors or legislators alone act as stakeholders. This makes it even more important for medical associations and medical societies to actively participate in this process.

## Framework for telemedicine in Germany

**Figure 1:**
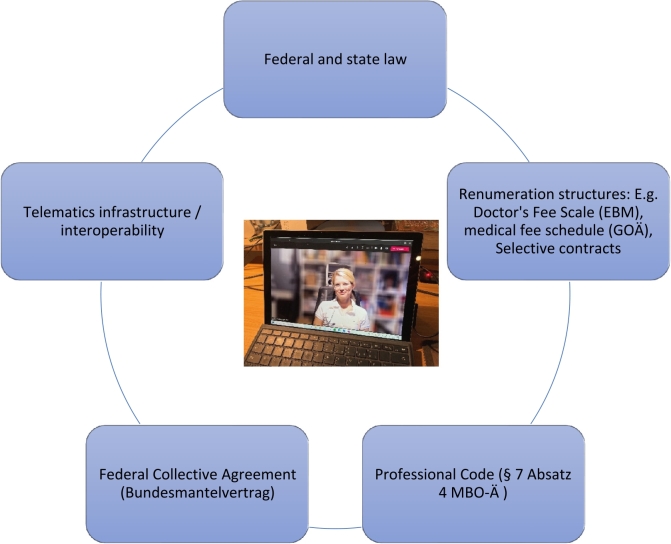
Framework for telemedical procedures.

Numerous regulations, (professional) legal issues, data protection, and technical aspects, as well as complex renumeration structures play a role in the application of telemedical procedures (Figure 1).

In the past, a lack of renumeration structures, uncertainties regarding the conformity of existing telemedicine software with data protection laws, questions regarding liability, and the ban on remote treatment in the professional code have been major obstacles to the use of telemedicine. In part driven by the Covid-19 pandemic, some of these issues have been addressed, including abolition of the ban on remote treatment, expansion of the renumeration options via the Doctor’s Fee Scale, and certification of video consultation providers by the National Association of Statutory Health Insurance Physicians (Kassenärztliche Bundesvereinigung; see https://www.kbv.de/html/videosprechstunde.php for further information). A platform that provides an overview of applications and projects that meet the legally required interoperability standards for telemedicine applications (www.vesta-gematik.de) has also been created. However, the use of telemedical methods in the field of medical genetics, particularly regarding genetic counseling, has not been adequately addressed and uncertainties regarding data protection and liability aspects remain. Another aspect in this context is the inadequate provision of broadband connections or 5G networks in many regions of Germany as a technical prerequisite for participation in telemedicine services.

To overcome these obstacles and to further promote and facilitate the use of telemedicine, the Federal Ministry of Health has presented a ministerial draft bill for a law on the digital modernization of care and nursing (DVPMG) in November 2020. The following aspects of the act are particularly relevant for medical genetics:

– Expansion of telemedicine: More attractive framework and improved renumeration for virtual consultations and teleconsultations, arrangement of telemedical consultations via the central appointment coordination (116 117).
– Unburdening of data protection regulations: Data protection impact assessment is already being carried out as part of the legislative process and is no longer the responsibility of the physicians.
– Expansion of the telematics infrastructure and improved user-friendliness: Creation of a digital identity for the insured persons and health service providers for secure identification (e. g., for virtual consultations), as well as a video communication and messenger service. An electronic patient file detached from the health card will be created.
– Promotion of interoperability by technical standardization and development of recommendations for the use of standards, profiles, and guidelines in eHealth.
  **Table 2:** Areas of telemedicine.

**Teleconsultations:** An exchange of information takes place between doctors (where appropriate also with the patient present) or doctors and other health professionals.
**Virtual consultations:** Doctor–patient contact usually takes place via video or other media without physical contact.
**Telediagnostics:** Medical reporting of examination results in spatial separation from the technical examination site (e. g., teleradiology).
**Telemonitoring:** Transmission of vital parameters or other patient-related data (predominantly in the case of chronic diseases) to the doctor, thereby enabling continuous monitoring and therapy adjustment.
– Creation of a digital National Health Platform for accessing health information by the Federal Ministry of Health.
– Improved coding of rare diseases: Mandatory use of Alpha-ID-SE and Orphanet identification numbers.

This ministerial draft bill has already been approved by the cabinet in January 2021 and is to come into force in May 2021. The act clearly shows the strong intention of the political players to advance digitization and the expansion of telemedicine. Its finalization will naturally also affect and promote the use of telemedicine in the field of medical genetics.

## Telemedical applications in practice

Table 2 shows the different areas of telemedicine.

A study from 2016 has shown that across Europe (lacking specific numbers for Germany) only 9 % of genetic professionals use telemedicine [6]. This figure will have risen by now. Nevertheless, the question arises as to which barriers are in the way of the widespread use of telemedicine in human genetics. Gordon et al. have identified discipline- and technology-specific challenges to integrating technical solutions into medical genetics [7]. Discipline-specific challenges include concerns that the caring, intensive, and intimate nature of counseling as well as the nonverbal aspects of communication cannot be well adapted via technology. In addition, there are concerns that the German system of Statutory Health Insurance Physicians could be undermined by the widespread use of telemedical genetic care, in which counseling is increasingly offered by internationally active large laboratories. Technology-specific challenges include a lack of interoperability, a decentralized nature of various technical solutions, or a lack of user-friendliness. In addition to the already mentioned improvement processes concerning the regulatory and technical framework, comprehensive medical informatics efforts to reduce technical barriers and improve technical and informatics standards are on their way. The “openEHR” foundation and community should be mentioned here, creating “a technology for e-health, consisting of open specifications, clinical models and software that can be used to create standards, and build information and interoperability solutions for healthcare” [8].

## Teleconsultation in medical genetics

Electronic consultation can be defined as asynchronous, consultative, provider-to-provider communication within a secure web-based platform and has been described as efficient and effective for a variety of subspecialties [9]. In Germany various concepts have been investigated in model projects under the Innovation Fund of the Federal Joint Committee highlighting the potential for implementation in standard care. Bhola et al. [9]. have been the first to describe the use of teleconsultation in the field of medical genetics in a pilot study in Eastern Ontario linking primary care providers (PCPs) and medical geneticists. PCPs were told to submit patient-specific clinical questions via a standardized form and attach additional information such as laboratory or other diagnostic results or patient images. Medical geneticists were able to respond by requesting additional information, providing recommendations, or recommending a referral for an in-person medical genetics appointment. In total, 111 consultations have been analyzed, roughly half addressing hereditary cancer (33 %) or genetic syndromes (21 %), while the other half comprised a large variability of topics, reflecting the broad spectrum of genetic counseling requests. The total of contemplated referrals via the PCPs prior to the electronic consultation was 72 %, compared to a total of 42 % planned referrals following the electronic consultation. Teleconsultations can therefore help to improve the allocation of scarce appointments in medical genetics and facilitate the initiation of genetic testing through the PCPs. A survey followed the completion of each electronic consultation and PCPs have reported that the electronic consultation service was of high value to themselves and to their patients.

Teleconsultation systems are also part of the European Reference Networks, which aim to tackle complex or rare diseases and conditions that require highly specialized treatment and a concentration of knowledge and resources. Via the Clinical Patient Management System (CMPS), a European Union-wide, cross-border, digital platform, relevant professionals are able to share challenging cases with international experts with the aim of seeking advice or sharing knowledge for the benefit of both patients and colleagues [10].

In Germany, an interesting concept of a teleconsultation platform is “Pädexpert” (www.paedexpert.de). Via this platform pediatricians in statutory health insurance or private practice can share findings with specialized colleagues (e. g., neuropediatricians) but also with other specialists, such as dermatologists, and submit patient-specific clinical questions in a structured environment via standardized anamnesis forms. Analogous to the abovementioned project from Canada, specialists can then respond by giving recommendations regarding the further course of action for the patient. The platform is compliant with data protection laws, and renumeration for the consultation service is provided via selective contracts with participating health insurance funds.

## Virtual consultation in medical genetics

Previously, virtual office hours were mostly offered within the framework of studies, although the Covid-19 pandemic has boosted the use of virtual consultations. High levels of patient satisfaction concerning system experience, information sharing, consumer focus, and overall satisfaction have been described in a review including 36 studies regarding virtual consultations from various specialities such as internal medicine, neurology, primary care, and gynecology [11]. In the field of medical genetics various studies have assessed the use of virtual consultations. The investigated counseling purposes have included cancer genetics, prenatal consultations, and presymptomatic cardiogenetic and oncogenetic consultations. From the counselees’ perspective, virtual consultations have been highly acceptable and virtual consultation has shown no inferiority in terms of patient satisfaction, anxiety, and perceived control [12], [13], [14], [15]. Studies regarding the perspective of the providers have also found high levels of satisfaction with virtual consultations, although some providers have felt a limited ability to engage in nonverbal communication and psychosocial support during a virtual consultation. There is a direct correlation between the technical quality of virtual consultation and provider satisfaction, highlighting the necessity of sufficient data connection and user-friendliness of the software used [16], [17]. Virtual consultations seem to be cost-effective and might contribute to process optimization [13]. Otten et al. have estimated a time reduction of 8 % and a cost reduction of 10–12 % [16].

Regarding the existing literature it should be kept in mind that measurement tools may need to be adapted for the evaluation of virtual consultations. Moreover, in previous studies, traditional genetic counseling was conducted via virtual communication and was compared to traditional in-person counseling. It should be further investigated to what extent, for example, the use of interactive learning tools or educational videos for the counselees changes the outcome of virtual counseling compared to conservative counseling.

## Telemedicine in the context of the medical genetics’ care landscape in Germany

The number of specialists for medical genetics in Germany is currently around 360 [18]. Of the human genetics outpatient clinics and practices, 88 % are located in cities with more than 100,000 inhabitants and 50 % in cities with more than 500,000 inhabitants [19]. The need for genetic counseling in Germany can be estimated at some 100,000 consultations per year, considering that more than 25,000 patients per year are newly diagnosed with a hereditary cancer [20], around 47,000 children per year are born with a genetic condition or a malformation [21], more than 3 million people are affected by a rare genetic disease, and 8,500 invasive prenatal examinations are carried out each year, with a subsequent need for genetic counseling and the resulting need of the care for family members at risk. Two consequences follow from these figures: The existing demand for genetic counseling can hardly be met by the available specialists and the population of structurally weak rural areas is virtually cut off from medical-genetic care. The next years will bring an increasing demand for genetic services. This is due to the rapid pace of laboratory technological advances, expansion of the personalized genomic medicine and genomic companion diagnostics, the discovery of new single-gene conditions, and the more complex nature of test results, which require specific expertise for their interpretation. As more genetic conditions are diagnosed definitively, there is also a concomitant cascade testing of family members [9]. Apart from efforts to increase the number of statutory health insurance medical geneticists and to strengthen education and training, telemedicine can be another instrument to address this issue. Teleconsultation can help to improve the allocation of scarce appointments in medical genetics, improve healthcare in rural areas, and contribute to significantly better preparation of genetic counseling in advance. The use of a teleconsultation platform allows precise documentation, which not only provides quality assurance, but also offers new approaches for health services research and offers new renumeration options by making the work of assessing requests from other specialists visible and comprehensible. Virtual consultations provide access to specialized medical genetic care for the rural population but can also facilitate access for the mobility-impaired or the chronically ill. The available studies do not give any reason for concerns regarding patient satisfaction or psychosocial outcomes. Currently, the biggest obstacle to the widespread use of virtual consultations, apart from technical and data protection concerns, certainly is the lack of renumeration structures in the field of medical genetics. Currently, renumeration for virtual consultations is only possible to an extremely limited extent within the framework of statutory and private health insurance in Germany. In shaping the improved renumeration of telemedicine demanded within the framework of the DVPMG, the medical genetics community in Germany represented by its professional societies therefore has decisive tasks to fulfill: There is a need for a clear definition of what areas of genetic counseling are suitable for video consultations. For example, the need for comprehensive physical examinations or the severity and scope of the diagnosis could play a role here. In a second step renumeration options for teleconsultations and virtual genetic consultations must be implemented timely to boost the usage of telemedicine in medical genetics. An important aspect in this context is to ensure that telemedicine services in medical genetics in Germany can and will only be offered by German medical licensed specialists/Statutory Health Insurance Physicians. An implementation of telemedicine is even more important as it must be assumed that the renumeration structures in the German healthcare system will be reformed in the coming years. The permission to offer certain diagnostics, procedures, and therapies will increasingly be linked to centers and an interdisciplinary indication. Especially in the field of personalized medicine, medical genetics must play an integral role and be part of these centers and interdisciplinary teams. A prerequisite for this, however, is that comprehensive medical genetic care is guaranteed in Germany. Telemedicine can help to ensure this care and, thus, strengthen the field in the future.

## Conclusion

Change is inevitable. The next few years will bring the technical solutions, interoperability, and political and regulatory framework for the widespread use of telemedicine in standard care in the field of medical genetics. Telemedicine offers great chances and has the potential to improve patient care and to contribute to the provision of comprehensive medical genetic care. The main risk for the medical genetics’ community is not being sufficiently active in shaping this change. Medical geneticists are characterized by a number of core competencies: Lifelong learning, the rapid adaptation of new scientific findings, the advancement of complex research and diagnostic methods, the expertise to interpret findings in a clinical context, the ability to translate complex scientific facts in an understandable way, and the assignment to offer psychosocial support for those seeking advice. These competences must be used and implemented in the field of telemedicine as well as to improve and maintain patient care and to consolidate medical genetics as an integral part of good medical care in the future.
